# Supplementary figures and images for: PMRT1, a Plasmodium-Specific Parasite Plasma Membrane Transporter, Is Essential for Asexual and Sexual Blood Stage Development
Source: mBio. 2022 Apr 11;13(2):e00623-22. doi: 10.1128/mbio.00623-22 (PMC9040750; doi:10.1128/mbio.00623-22)

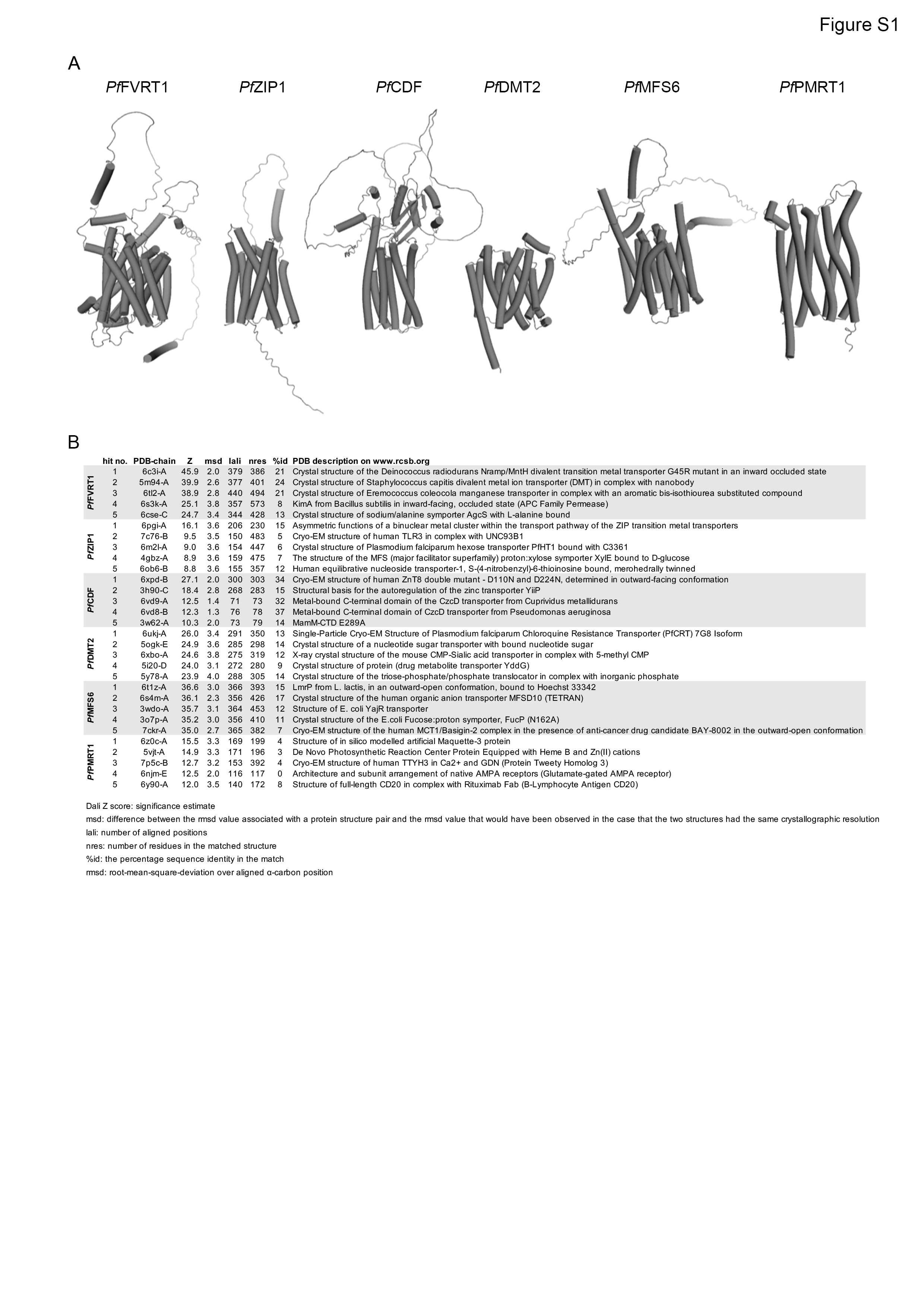

Supplement: FIG S1 [file mbio.00623-22-sf001.tif]

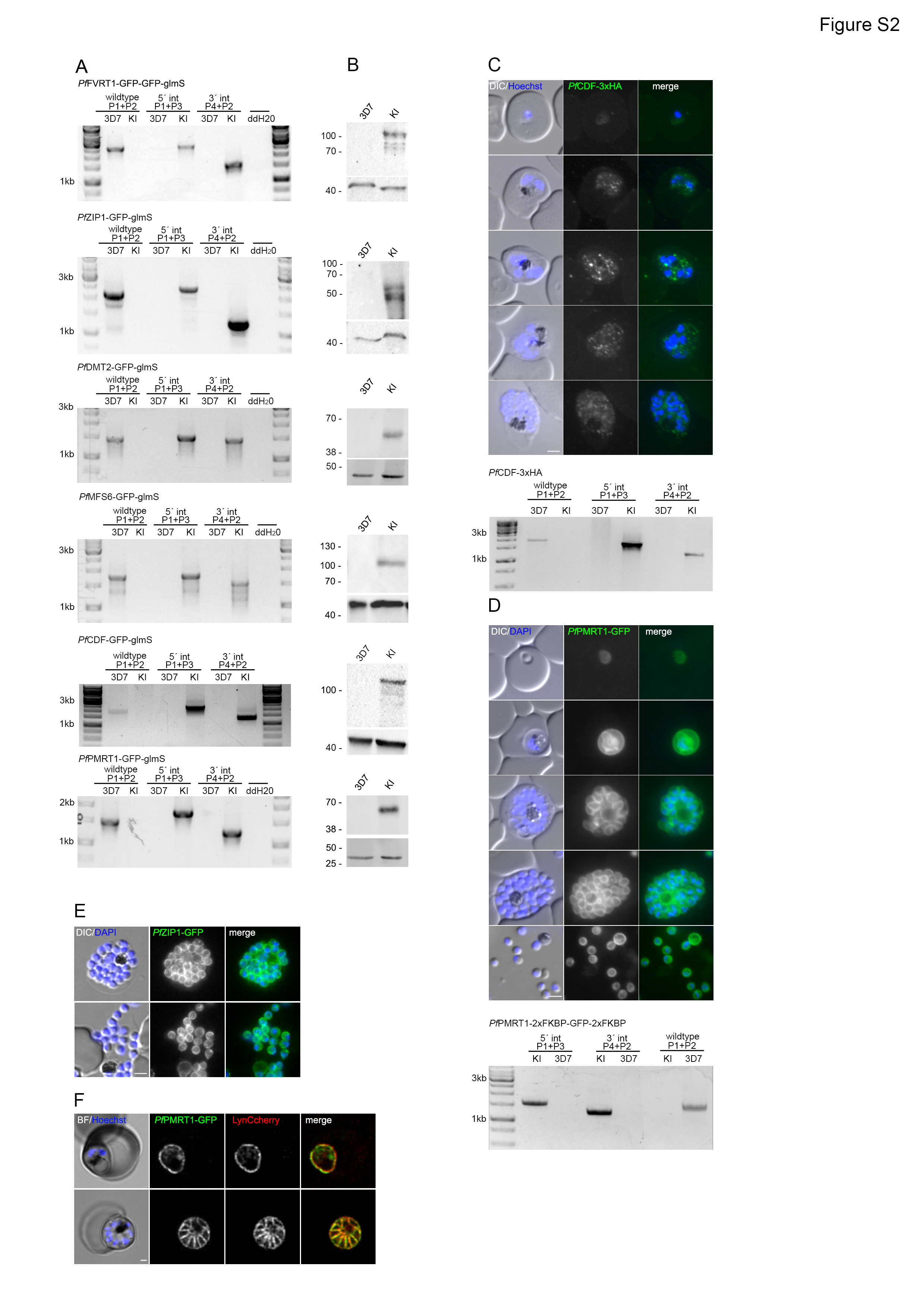

Supplement: FIG S2 [file mbio.00623-22-sf002.tif]

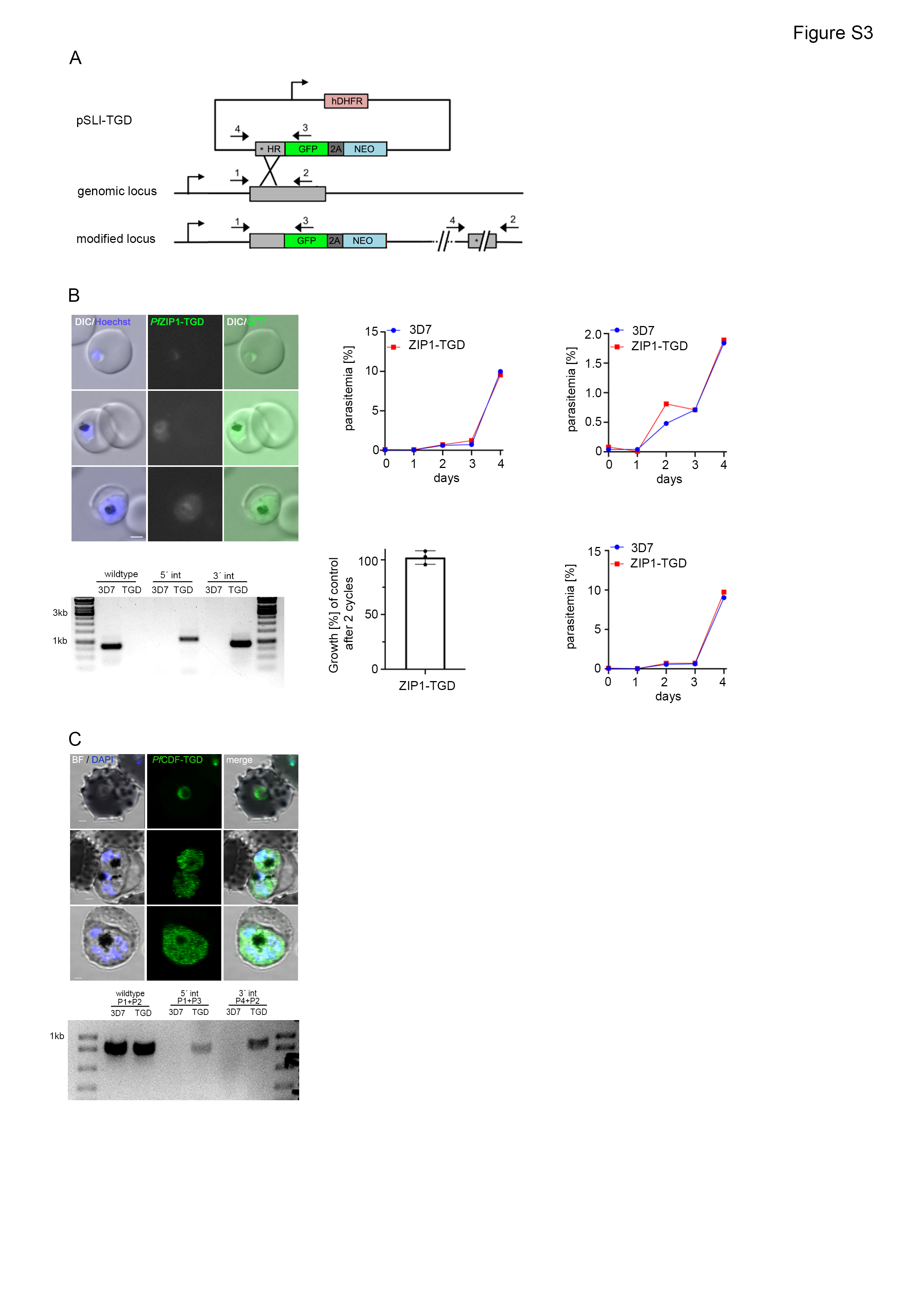

Supplement: FIG S3 [file mbio.00623-22-sf003.tif]

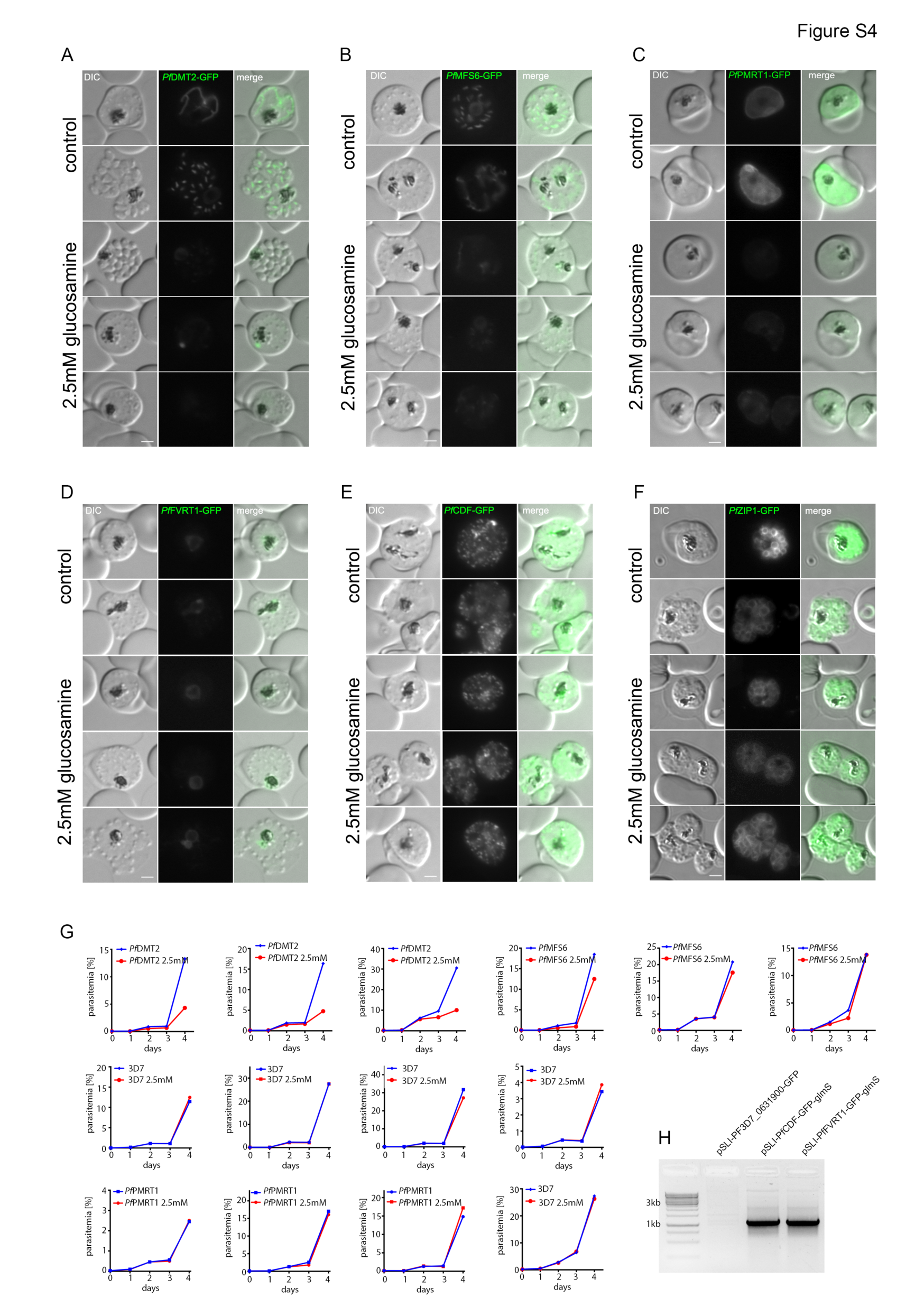

Supplement: FIG S4 [file mbio.00623-22-sf004.tif]

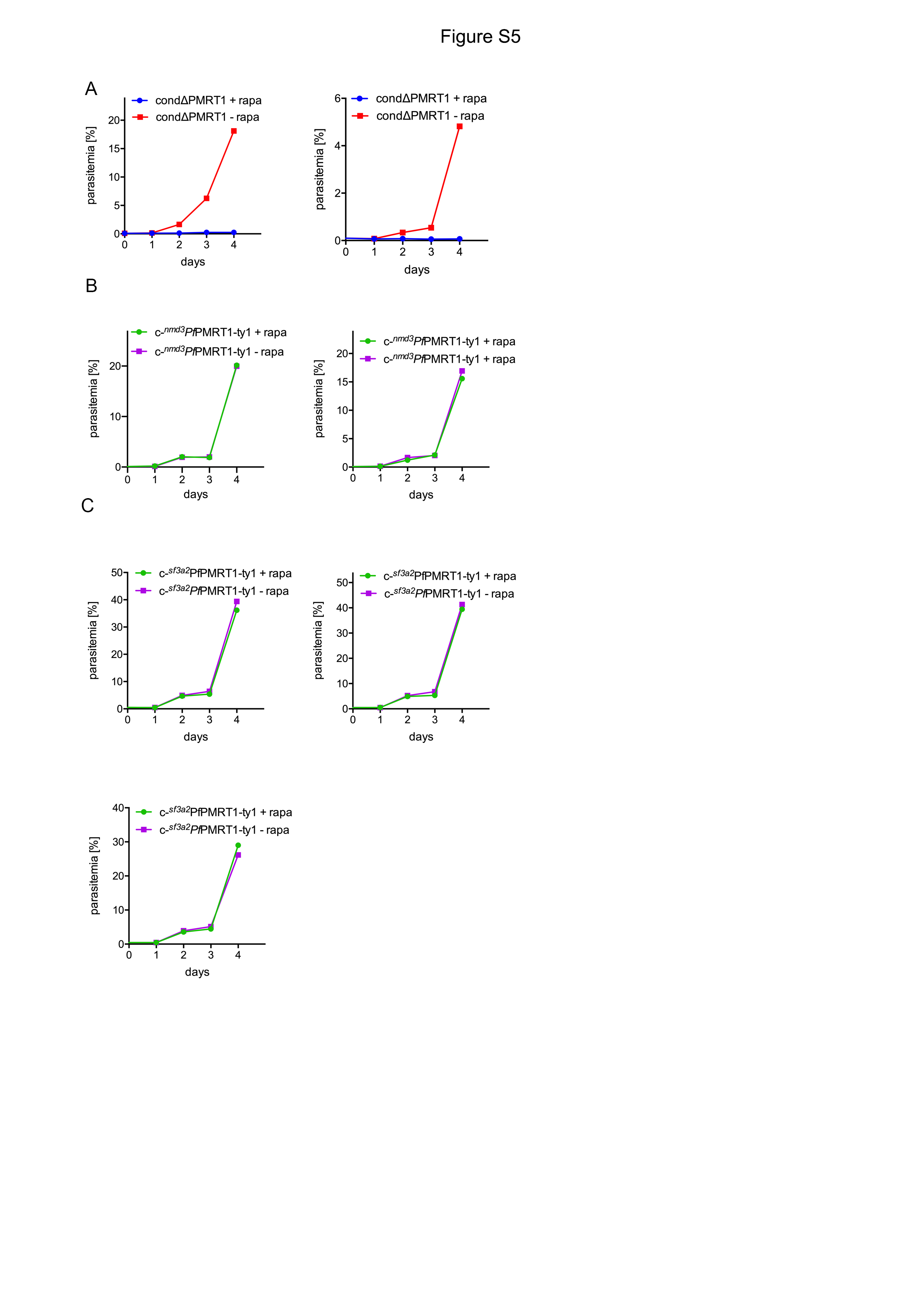

Supplement: FIG S5 [file mbio.00623-22-sf005.tif]

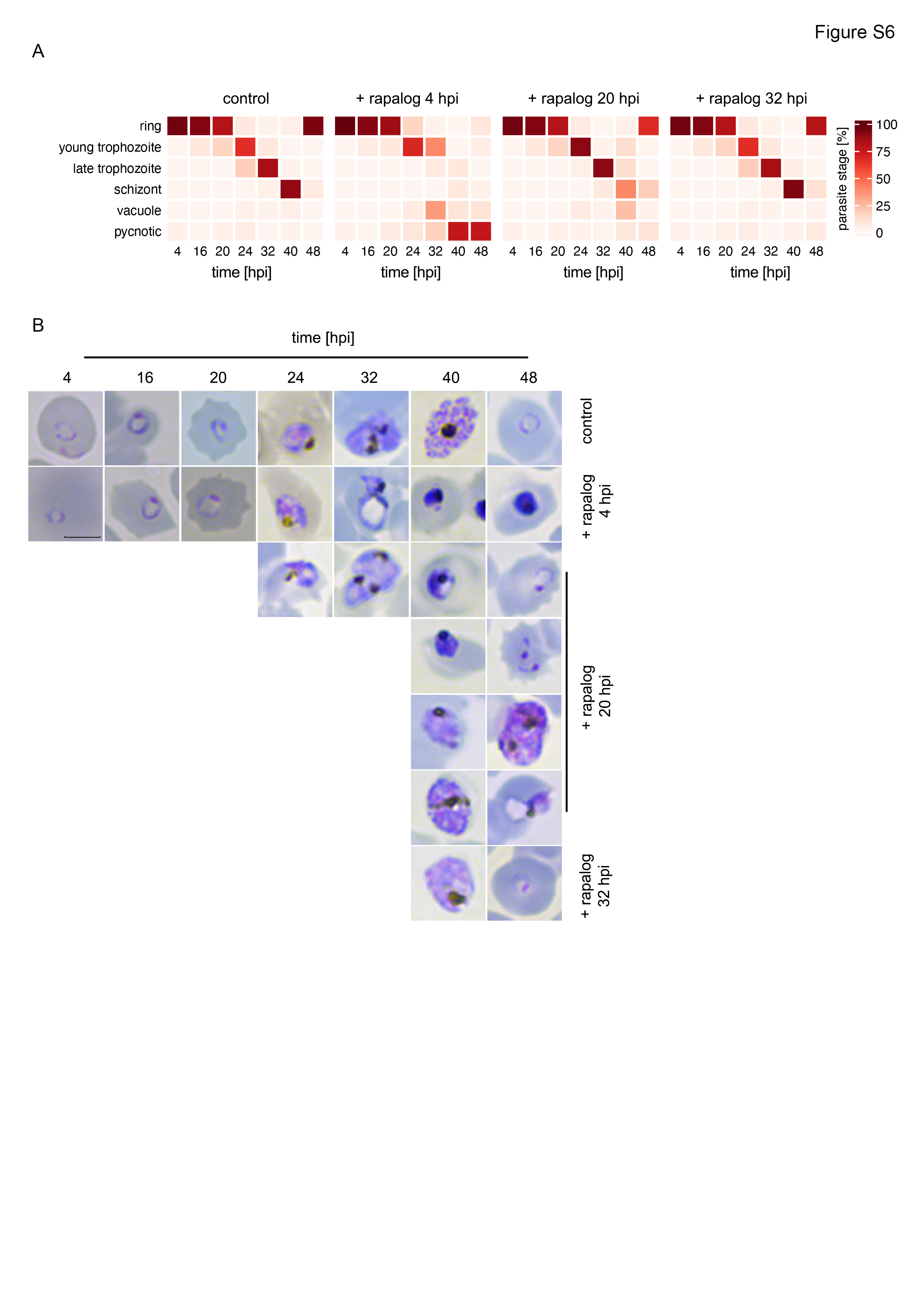

Supplement: FIG S6 [file mbio.00623-22-sf006.tif]

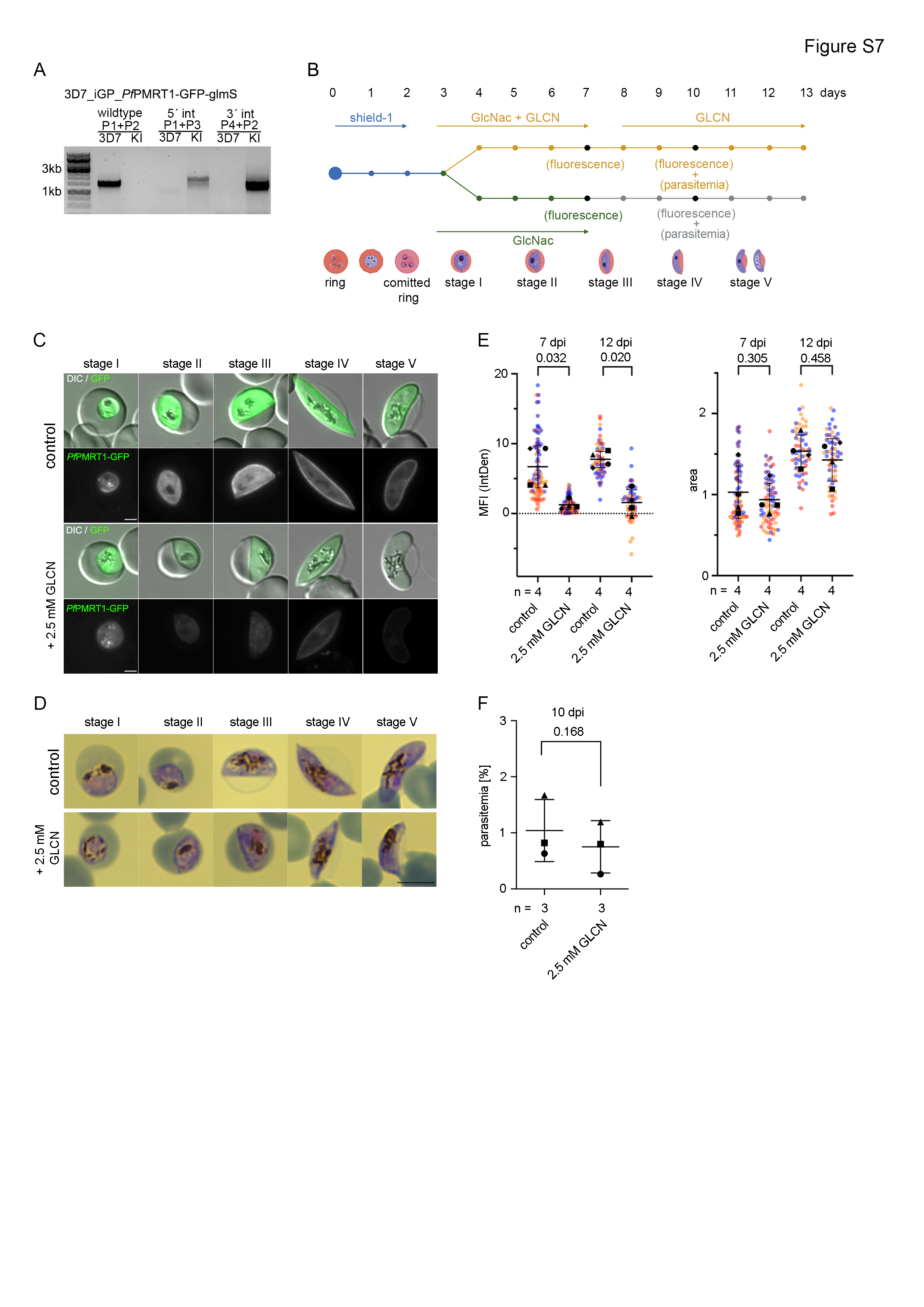

Supplement: FIG S7 [file mbio.00623-22-sf007.tif]

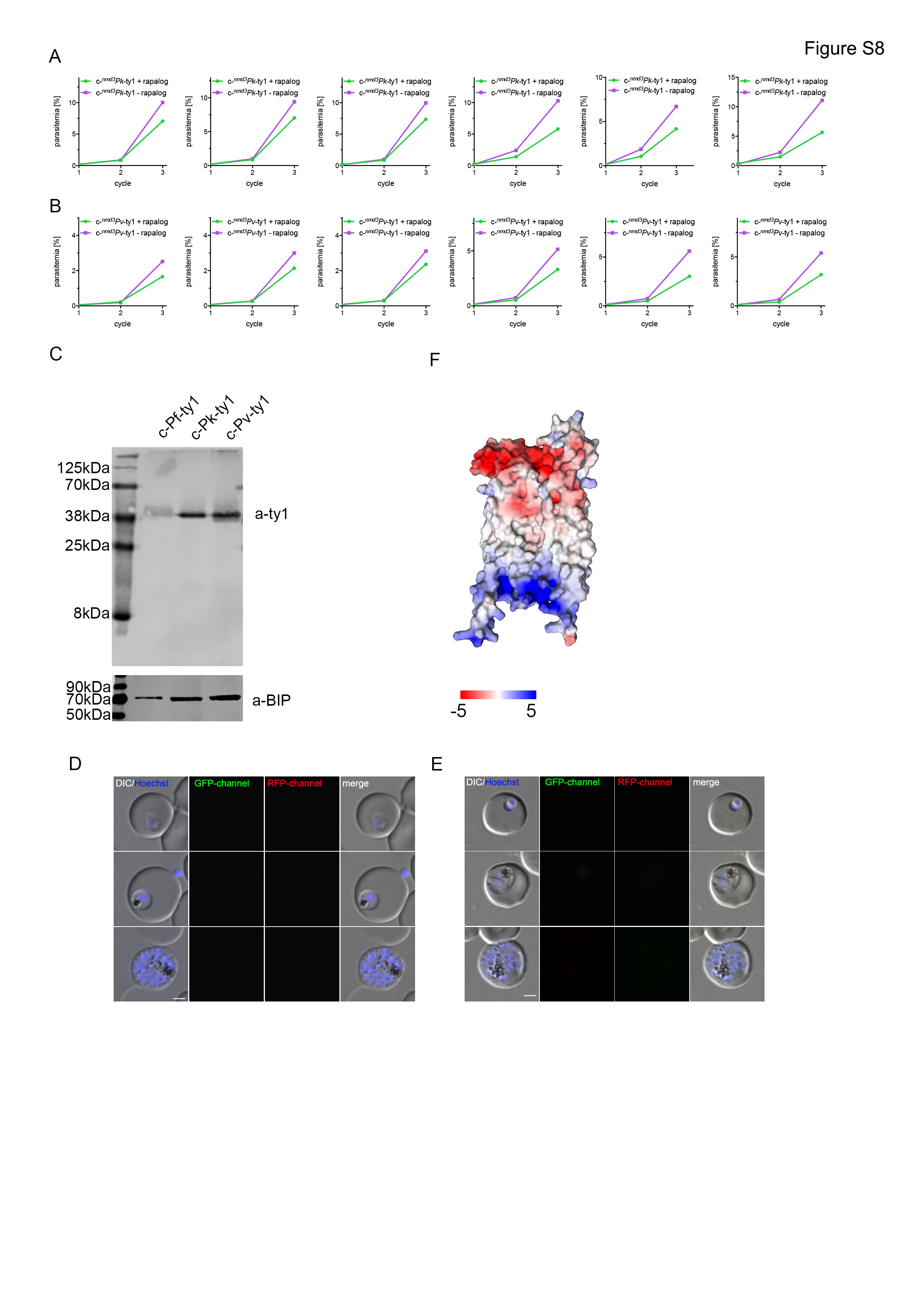

Supplement: FIG S8 [file mbio.00623-22-sf008.tif]
