# Supplementary material for: PMRT1, a Plasmodium-Specific Parasite Plasma Membrane Transporter, Is Essential for Asexual and Sexual Blood Stage Development
Source: mBio. 2022 Apr 11;13(2):e00623-22. doi: 10.1128/mbio.00623-22 (PMC9040750; doi:10.1128/mbio.00623-22)
Supplement: TABLE S1 [file mbio.00623-22-st001.pdf]

Table S1: Oligonucleotides **(A)** and plasmids **(B)** used in this study.

A

internal #	Target or Primer name	Sequence	Purpose
284	PF3D7_0716900 rv	GGGacgcgtTAACATCAATTTTGCTTTTTTGGG	cloning GFP-glmS
285	PF3D7_0716900 fw	GGGgcggccgctaaGGGTGTGGTAATTCAAGTACG	
280	PF3D7_0523800 rv	GGGacgcgtATTTCGTTGAATATAATTTTTTTTAATTG	
281	PF3D7_0523800 fw	GGGgcggccgctaaGTTTTCTTGTTGGTATTTTAGCTG	
282	PF3D7_0609100 rv	GGGacgcgtATGATTATGACCATGATCATGATC	
283	PF3D7_0609100 fw	GGGgcggccgctaaGGATAGCAGGTGTTACGGTTTCTTTATC	
288	PF3D7_1440800 rv	GGGacgcgtATTAGTAATAGAATTTTTCATCTTG	
289	PF3D7_1440800 fw	GGGgcggccgctaaCATTTGCTTCAAATTTGATGAG	
290	Pf3D7_1135300 rv	GGGacgcgtAGAAGTTTTTGGGGCATATTTCTTTG	
338	Pf3D7_0715900 (11)	GGGgcggccgctaaCTGGAAATAAAATAGATGGAACGTCTTG	
339	Pf3D7_0715900 (11)	GGGacgcgtAGTATCCCCTTTCAATGTGGAAC	
291	Pf3D7_1135300 fw	GGGgcggccgctaaGTTATATTATAAAAGGATGATTGG	
315	PF3D7_0523800 (1) TGD fw	GCGGCCGCTAAAGGAGCACTAAAGGCCAAAGGAAGT	cloning TGD
316	PF3D7_0523800 (1) TGD rv	acgcgtAATAATGTCATCCTTTTCATTATTAATATT	
317	PF3D7_0609100 (2) TGD fw	GCGGCCGCTAAGATTTGTTATTTGCAAAAATAATTTGTATT	
318	PF3D7_0609100 (2) TGD rv	acgcgtATGTctataaaaaaaataaatcacatac	
319	PF3D7_0716900 (3) TGD fw	GCGGCCGCTAAAAAAATGAAATTATATTTTGTACGACATTC	
320	PF3D7_0716900 (3) TGD rv	acgcgtGGCAATACCAAAGGTAATTAATAAAATTCC	
323	PF3D7_1440800 (5) TGD fw	GCGGCCGCTAAACAATGTTTAAGGATGATGAAAATAATTTT	
324	PF3D7_1440800 (5) TGD rv	acgcgtTGTATTAACAACAACTATAAATACAGAACT	
	Pf3D7_0715900 SLI-Tdg F	GGTGCGGCCGCGTGGATAAGATGTCGCGTTTG	
	Pf3D7_0715900 SLI-Tgd R	GGTACGCGTGTGTCCTACATTTTTATCATCTTCCTC	
	PF3D7_1135300 – fw NotI TGD	CTCGgcggccgctaaAAAAGTATGATCTCTGGAATATCCAG	
	PF3D7_1135300 – rv MluI TGD	TCCTacgcgtATTTATAAAGAGATCTGTTTTATTATC	
	PF3D7_1135300 fw NotI loxP	CTCGGCGGCCGCCCGGTAAATCTCTGGAATATCCAGCAAAATTG	cloning 1135300 loxp
	PF3D7_1135300 rv SpeI loxP:	TCCTACTAGTATTTATAAAGAGATCTGTTTTATTATC	
	PF3D7_1135300 fw AvrII loxP:	CTCGCCTAGGATGAAGTCAATGATAAGCGGTATTAG	
	PF3D7_1135300 rv XmaI loxP:	TCCTCCCGGGGCTTGTCTTAGGTGCGTACTTTTTAC	
294	PF3D7_0609100 int_fw	GGTAGTAACAACCTTTGGTTGTTTTATTCC	integration check PCR
295	PF3D7_0609100 int_rv	tatgtggaaggttaattaaatggacaaggg	
296	PF3D7_0716900 int_fw	GAAATTATATTTTGTACGACATTCATACTA	
298	PF3D7_1440800 int_fw	GCACAGAACATTTAAGAAGCAATGATTTTA	
299	PF3D7_1440800 int_rv	caacttgactagccaaaatgttggttctgg	
300	PF3D7_1135300 int_fw	GATCTCTGGAATATCCAGCAAAATTGTTG	
302	PF3D7_0716900 int_rv	atatttatttattttgaaccgataagctag	
305	PF3D7_1135300 int_rv	gccatatatattatacatattaaatataaagac	
308	PF3D7_0523800 int_fw	GGCCAAAGGAAGTTGGCTAACGGGGTGGTAG	
309	PF3D7_0523800 int_rv	gtgttcattcatttaccttttgaatgg	
369	TGD (PF3D7_0609100) int check fw	atatatgttttaaagcttcaaaatg	
370	TGD (PF3D7_0609100) int check rv	gaggagaagcaaaaacgaaagttaac	
392	TGD (Pf3D7_0715900) int check fw	CCCACCGAAATGAACTCTTCGTTGC	
393	TGD (Pf3D7_0715900) int check rv	CTTTACCTTTAGAGGAGGAATTATTAG	
394	SLI TGD it fw Adelaide	gtggaattgtgagcggataac	
327	Pf3D7_1135300 compl	ctcgagATGAAGTCAATGATAAGCGG	cloning complementation constrcuts
328	Pf3D7_1135300 compl	cctaggGCTTGTCTTAGGTGCGTACT	
553	PKNH_0933400 fw 553	GGGctcgagATGAAGGGAACGTACGTAG	

554	PKNH_0933400 rv 554	GGGcctaggCACCGCCTTCGAGGCGTAC	
555	PVP01_0936100 fw 555	GGGctcgagATGAAGGGAACGTACGTCG	
556	PVP01_0936100 rv 556	GGGcctaggCACCGCCTTCGAGGCGTAC	
p37	Pf3D7_1135300 fw 5' (TKo)	GATTTTGATATATGATTATAGGATAG	excision PCR primer
	Neo 40 rv	CGAATAGCCTCTCCACCCAAG	
	Pf3D7_1135300 fw NotI	CTCGgcggccgctaaTTATTATAAATCATATAATAAAATAAATG	cloning 1135300-2xFKBP-GFP
	Pf3D7_1135300 rv AvrII	TCCTcctaggAGAAGTTTTTGGGGCATATTTCTTTG	
443	Pf3D7_0715900 (11) KpnI	GggtaccAGTATCCCCTTTCAATGTGGAAC	cloning 0715900-3xHA
	GFP 633 fw	GCCCTTTCGAAAGATCCC	confirmatory PCR
	glmS rv	GATTTCTCTTTGTTCAAGGAGTCACC	

B

pSLI-*Pf*FVRT1-GFP-glmS	this study
pSLI-*Pf*ZIP1-GFP-glmS	this study
pSLI-*Pf*CDF-GFP-glmS	this study
pSLI-*Pf*DMT2-GFP-glmS	this study
pSLI-*Pf*MFS6-GFP-glmS	this study
pSLI-*Pf*PMRT1-GFP-glmS	this study
pSLI-*Pf*FVRT1-GFP-TGD	this study
pSLI-*Pf*ZIP1-GFP-TGD	this study
pSLI-*Pf*CDF-GFP-TGD	this study
pSLI-*Pf*DMT2-GFP-TGD	this study
pSLI-*Pf*MFS6-GFP-TGD	this study
pSLI-*Pf*PMRT1-GFP-TGD	this study
pSLI-*Pf*CDF-3xHA	this study
pSLI-*Pf*PMRT1-2xFKBP-GFP	this study
P40PX-mCherry	Jonscher et al. 2018 (44)
pLyn-FRB-mCherry	Birnbaum et al. 2017 (41)
pACP-mCherry	Birnbaum et al. 2020 (46)
pARL-^{ama1}ARO-mCherry	Cabrera et al. 2012 (49)
pARL-^{ama1}AMA1-mCherry	Wichers et al. 2021 (51)
pSLI-*Pf*PMRT1-loxP	this study
pARL-^{nmd3}*Pf*PMRT1-ty1	this study
pARL-^{sf3a2}*Pf*PMRT1-ty1	this study
pHcamGDV1-GFP-DD-yDHODH	this study
pSkip-Flox	Birnbaum et al. 2017 (41)
pARL- ^{nmd3}*Pv*PMRT1-ty1	this study
pARL- ^{nmd3}*Pk*PMRT1-ty1	this study
